# Deer presence rather than abundance determines the population density of the sheep tick, *Ixodes ricinus*, in Dutch forests

**DOI:** 10.1186/s13071-017-2370-7

**Published:** 2017-09-19

**Authors:** Tim R. Hofmeester, Hein Sprong, Patrick A. Jansen, Herbert H. T. Prins, Sipke E. van Wieren

**Affiliations:** 1 Resource Ecology Group, Wageningen University, Droevendaalsesteeg 3a, 6708PB Wageningen, The Netherlands; 2 Centre for Infectious Disease Control Netherlands, National Institute for Public Health and Environment, Antonie van Leeuwenhoeklaan 9, 3721 MA Bilthoven, The Netherlands; 3 Center for Tropical Forest Science, Smithsonian Tropical Research Institute, Balboa, Ancon, Republic of Panama

**Keywords:** *Capreolus capreolus*, *Cervus elaphus*, *Dama dama*, Deer management, Passage rate, Reproduction host, Tick density

## Abstract

**Background:**

Understanding which factors drive population densities of disease vectors is an important step in assessing disease risk. We tested the hypothesis that the density of ticks from the *Ixodes ricinus* complex, which are important vectors for tick-borne diseases, is determined by the density of deer, as adults of these ticks mainly feed on deer.

**Methods:**

We performed a cross-sectional study to investigate *I. ricinus* density across 20 forest plots in the Netherlands that ranged widely in deer availability to ticks, and performed a deer-exclosure experiment in four pairs of 1 ha forest plots in a separate site.

**Results:**

*Ixodes ricinus* from all stages were more abundant in plots with deer (*n* = 17) than in plots without deer (*n* = 3). Where deer were present, the density of ticks did not increase with the abundance of deer. Experimental exclosure of deer reduced nymph density by 66% and adult density by 32% within a timeframe of two years.

**Conclusions:**

In this study, deer presence rather than abundance explained the density of *I. ricinus*. This is in contrast to previous studies and might be related to the relatively high host-species richness in Dutch forests. This means that reduction of the risk of acquiring a tick bite would require the complete elimination of deer in species rich forests. The fact that small exclosures (< 1 ha) substantially reduced *I. ricinus* densities suggests that fencing can be used to reduce tick-borne disease risk in areas with high recreational pressure.

**Electronic supplementary material:**

The online version of this article (10.1186/s13071-017-2370-7) contains supplementary material, which is available to authorized users.

## Background

Ticks are important vectors for diseases such as Lyme borreliosis, Mediterranean spotted fever and tick-borne encephalitis [1]. Understanding which factors influence population densities of ticks is an important step in identifying the causes for elevated disease risk [2]. Many tick species spend part of their life in the vegetation searching for a host from which they must acquire blood in order to survive and reproduce [3]. Because the number of bloodmeal hosts available in the environment determines the likelihood of a tick finding a host [4], the presence and density of hosts is considered an important determinant of tick density.

Ticks of the *Ixodes ricinus* complex have three active life stages, larva, nymph and adult [1, 5, 6], all of which search for a host by questing in the vegetation, but do so at different heights, probably related to differences in host preference [7]. In typical forested areas, most larvae parasitize small mammals, while most adults parasitize deer [8]. It is widely assumed that deer are essential hosts in the life-cycle of ticks from the *I. ricinus* complex, hence that disease risk can be controlled by reducing deer densities. This assumption is supported by several studies that found a strong correlation of tick density with deer presence and density [9–11]. Several other studies, however, found that deer exclusion (by fencing) and deer culling did not always reduce tick densities [12, 13]. Thus, it is still unclear whether and how management of deer populations reduces tick densities.

Three modelling studies that took the complex life-cycle of ticks into account suggested that the relationship between deer and tick densities is non-linear, and different for the different stages [14–16]. Van Buskirk & Ostfeld [16], for example, modelled how nymph densities of *I. scapularis*, the black-legged tick, responded to differences in densities of hosts for larvae and adults, and found that the density of hosts for adults was limiting nymph density only at very low host densities, where the availability of hosts for larvae then became limiting. Thus, in sites where other host species than deer are the main hosts for immature stages, the density of nymphs and adults appears to increase with deer density according to a non-linear threshold relationship, rather than the linear relationship used in most studies (e.g. [9, 17]). Furthermore, Van Buskirk & Ostfeld [16] suggested that the threshold host density for adult ticks is close to zero. Field tests considering a wide range of deer densities including zero are needed to test these predictions.

Here, we empirically assess the abundance relationship between *I. ricinus*, the sheep tick, and three species of deer, roe deer (*Capreolus capreolus*), red deer (*Cervus elaphus*) and fallow deer (*Dama dama*), in forests in the Netherlands. We used a cross-sectional study across 20 forest plots that ranged from having no deer at all to having very high deer densities. We tested the predictions that (i) *I. ricinus* densities of all stages are low in areas where deer are absent compared to areas where deer are present, and that (ii) where deer are present, the number of questing *I. ricinus* increases linearly with deer density. Furthermore, we compared tick densities between four pairs of experimental deer exclosures and control plots at one site to test the assumption that (iii) deer are essential hosts for *I. ricinus*.

## Methods

### Study sites

**Fig. 1:**
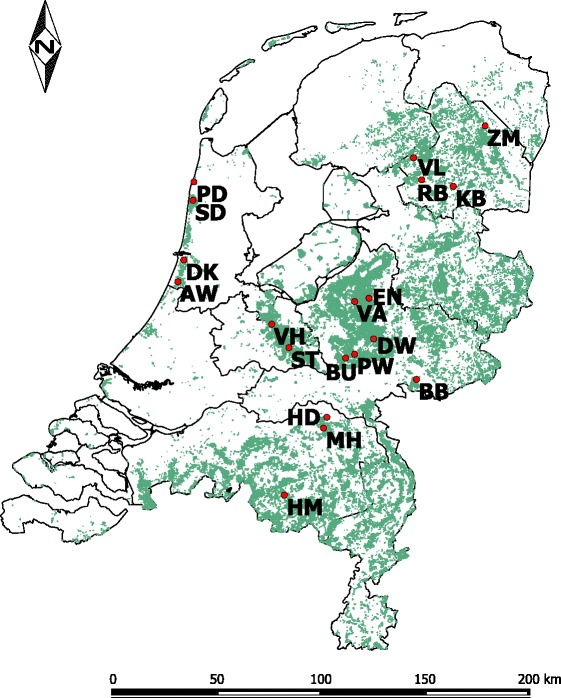
Map of the Netherlands with the 20 plots of the cross-sectional study. Forested areas are shown in green, provincial borders in black. *Abbreviations*: AW, Amsterdamse Waterleiding Duinen; BB, Bergherbos; BU, Buunderkamp; DK, Duin en Kruidberg; DW, Deelerwoud; EN, Enkhout (two plots including exclosure); HD, Herperduin; HM, Halfmijl; KB, Kremboong; MH, Maashorst; PD, Pettemerduinen; PW, Planken Wambuis; RB, Landgoed Rheebruggen; SD, Schoorlse duinen; ST, Stameren; VA, Valenberg; VH, Vijverhof; VL, Landgoed Vledderhof; ZM, Zwanemeerbos

The cross-sectional study encompassed twenty 1 ha plots in nineteen forested areas in the Netherlands (Additional file 1: Table S1), which were > 5 km apart (Fig. 1). We sampled eleven plots in 2013 and nine in 2014. All plots were located within forested areas with pedunculate oak (*Quercus robur*), Scots pine (*Pinus sylvestris*), or a combination of these as dominant tree species (Additional file 1: Table S1), and selected based on distribution patterns of deer in the Netherlands [18]. One study area, Enkhout, had two plots that were just 150 m apart, but one of these was located in a 3 ha stand fenced three years prior to the study, that thus had no ungulates.

To experimentally remove deer, we placed four exclosures in a forested area near Apeldoorn, the Netherlands (52°14′N, 5°55′E) following a Before-After Control-Impact design [19]. The fences (2.2 m high) were erected in May 2013 and included 0.61–0.78 ha of mixed forest with an understory dominated by blueberry (*Vaccinium myrtillus*). Each exclosure had a control plot with a similar vegetation and forest structure, *c.*100 m away from the exclosure. These plots were sampled twice, when the exclosures were build and two years later, in 2015.

### Availability of deer

We measured the availability of deer as hosts to ticks as described in Hofmeester et al. [20]. In short, we measured passage rate per deer species between March and November, the main activity season of *I. ricinus* in the Netherlands [21] by running camera traps (HC500, Reconyx Inc., Holmen, WI, USA) at 18 random points per plot following the protocol described by Hofmeester et al. [22], resulting in an average of 504 sampling days per plot (Additional file 1: Table S1). We used the estimates of effective detection distance given in Hofmeester et al. [20] to estimate passage rates per species per camera trap deployment. For each plot, we calculated an average passage rate (m^-1^·d^-1^) per species by using the arithmetic mean. To be able to test for a correlation with total deer availability, we summed the passage rates of all deer species to determine the availability of all deer to ticks.

For the experimental study, we used camera traps during July-November 2015 to assess the presence or absence of deer in all plots. In each plot, 20 locations were sampled for one week each, using two cameras per plot with an inter-camera spacing of > 30 m. Camera settings and placement were identical to the cross-sectional study [22].

### Tick density

For the cross-sectional study, each of the 20 plots was visited six times during April-September, i.e. once every four weeks, to collect ticks by blanket-dragging twenty transects of 10 m length using a 1 m^2^ cotton cloth [23]. We dragged for ticks only on dry days that had an air temperature > 10 °C and on dry vegetation [4, 7], and we minimized variation in weather conditions between plots by visiting all plots within five days. After each 10-m drag we counted all *I. ricinus* larvae, nymphs and adults on the cloth, and used these numbers to determine an average tick density (per 100 m^2^) for each life stage per plot. Nymphs and adults were identified to species using an established identification key [24]. As all but two were *I. ricinus*, we assumed that all larvae found were also *I. ricinus*. This assumption is safe as a previous study in the Netherlands collected only larvae of *I. ricinus* with drag sampling [21].

For the experimental study, we estimated tick density in each of the eight plots four times: In May and August 2013, just after the fences were placed, and again in May and August 2015, two years after fencing. During each visit, 15 transects of 10 m were dragged, and all *I. ricinus* nymphs and adults on the cloth counted, to estimate the average tick density (per 100 m^2^) for each stage for both years. We did not collect larvae.

### Statistical analysis

Predictions were tested in R 3.2.2 [25], using generalized linear mixed models (GLMM) with a negative binomial distribution and log link function, as implemented in the *glmmADMB* package [26, 27]. For the cross-sectional study, we performed two separate tests. First, we tested for a difference in *I. ricinus* density, by life stage, between plots with and without deer. Secondly, we used two models to test for a correlation of *I. ricinus* density, by life stage, with the availability of fallow deer, red deer and roe deer (model 1), and of all deer species combined (model 2), using only the 17 plots with deer presence. Passage rates were standardized by extracting the mean and dividing by two standard deviations [28]. We allowed the intercept of all models to differ between vegetation types to correct for possible bias of the vegetation on the density estimates, and added year as a fixed factor to correct for possible differences in questing tick densities between the years.

For the experimental study, we tested for differences in nymph and adult tick density between control plots and exclosures, and between years using a GLMM with a negative binomial distribution and log link. The GLMM included both factors and an interaction term with a random intercept per plot nested within site. We included the random intercept to correct for repeated measurements within each plot (one measurement in 2013 and one in 2015) and the paired design (one exclosure and one control plot within each site).

## Results

### Cross-sectional study

Three plots (the Enkhout exclosure, Pettemerduinen and Schoorlse Duinen) lacked deer. In the other 17 plots, passage rates of deer ranged from 0.01 to 0.84 m^-1^·d^-1^ (Additional file 2: Table S2). Roe deer (*Capreolus capreolus*) were present in 15 plots with passage rates ranging six-fold, from 0.01 to 0.06 m^-1^·d^-1^, fallow deer (*Dama dama*) were present in five plots, with passage rates ranging more than 800-fold, from 0.001 to 0.84 m^-1^·d^-1^, and red deer (*Cervus elaphus*) were present in four plots with passage rates ranging four-fold, from 0.01 to 0.04 m^-1^·d^-1^ (Additional file 2: Table S2).

**Fig. 2:**
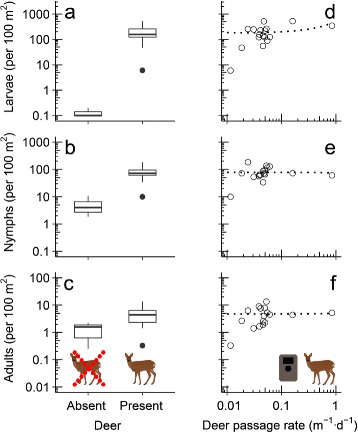
Relationship between the density of *Ixodes ricinus* and the availability of deer. Tick density differed significantly (*P* < 0.05) between 17 forest plots with deer and 3 plots without (**a**-**c**), but did not significantly increase with the availability of deer across the 17 plots with deer (**d**-**f**), for larvae (**a**, **d**), nymphs (**b**, **e**) and adults (**c**, **f**). Dotted lines represent the generalized linear mixed model fits for non-significant models. +0.1 was added to the larval densities in the graph to overcome problems due to zeroes and the logarithmic scale on the y-axis

A total of 38,535 larvae, 16,617 nymphs and 1019 adults of *Ixodes ricinus* were counted and collected in the 20 plots. The density of larvae ranged across plots from 0 to 517 per 100 m^2^, nymph density from 2 to 183 per 100 m^2^ and adult density from 0.3 to 13 per 100 m^2^ (Additional file 2: Table S2). Densities of larvae were on average 99.99% lower (and nearly zero) in the three plots without deer than in the 17 plots with deer (GLMM: β = 8.7, *P* = 0.007; Fig. 2a) and did not differ significantly between the years (β = 0.3, *P* = 0.43). Nymph densities were on average 93.1% lower in plots without deer than in plots with deer (β = 2.7, *P* < 0.001, Fig. 2c) and adult densities on average 71.4% lower (β = 1.2, *P* = 0.04, Fig. 2e), which agrees with prediction 1. Densities of nymphs (β = -0.2, *P* = 0.42) and adults (β = 0.2, *P* = 0.45) were not significantly different between the years. However, in plots that had at least one species of deer, *I. ricinus* densities were not correlated with the availability of specific deer species or all deer combined (Table 1; Fig. 2), which disagrees with prediction 2.

**Table 1:** Relationship between the density of *Ixodes ricinus*, by life stage, and deer activity, across 17 plots that had at least one species of deer

Model	Deer species	Larva		Nymph		Adult	
		β	*P*	β	*P*	β	*P*
1	Fallow deer	0.27	0.64	0.15	0.63	0.21	0.58
	Red deer	-0.27	0.66	0.49	0.17	0.64	0.14
	Roe deer	-0.21	0.74	0.27	0.46	0.15	0.75
2	All deer	0.42	0.35	-0.02	0.95	0.03	0.93

Correlation coefficient (β) and *P*-value for correlations between *I. ricinus* density of the three different life stages and availability of fallow deer (*Dama dama*), red deer (*Cervus elaphus*) and roe deer (*Capreolus capreolus*; model 1) or all deer species combined (model 2) as obtained with generalized linear mixed models with a negative binomial distribution and log link with a random intercept per vegetation type

### Deer exclosure study

**Fig. 3:**
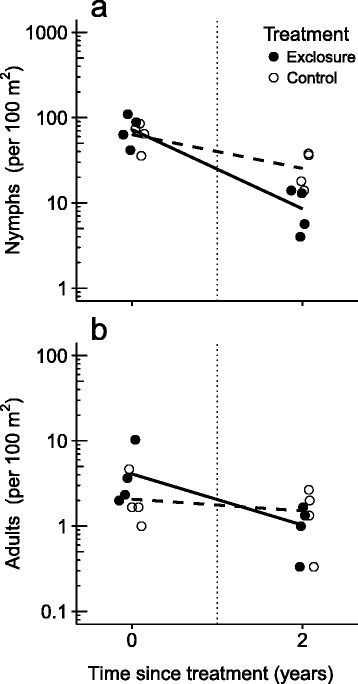
Effect of small deer exclosures (< 1 ha) on *Ixodes ricinus* density. Deer exclusion reduced the density of questing nymphs (**a**) and adults (**b**) over two years in four paired plots using a Before-After Control-Impact design. Solid lines show the generalized linear mixed model fits for exclosures, and dotted lines the model fits for control plots

Camera traps detected roe deer and red deer in all control plots, and photographed none in any of the exclosures, confirming that the exclosures were effective in excluding deer. We collected a total of 1691 nymphs and 82 adults of *I. ricinus* in 2013 and 429 nymphs and 32 adults in 2015 (Additional file 2: Table S3). There was no initial difference in nymph densities between exclosures and controls (GLMM: difference = 0.1, *P* = 0.57). Two years later, nymph densities were significantly lower in all plots (difference = -1.5, *P* < 0.001), and nymph densities were significantly lower (66%) in exclosures than in control plots (difference = -1.1, *P* < 0.001; Fig. 3a). Exclosures had higher initial adult densities than controls (difference = 0.7, *P* = 0.04). Two years later, adult densities had significantly reduced in exclosures (difference = -1.4, *P* < 0.001), but not in control plots (difference = -0.4, *P* = 0.41), resulting in lower (32%) adult density in exclosures than in control plots (Fig. 3b). These results support prediction 3.

## Discussion

Deer are widely considered the most important determinant of the densities of ticks in the *Ixodes ricinus* complex (e.g. [5]), but the shape of the abundance relationship remains poorly known. We studied the relationship between the availability of deer and tick density in a cross-sectional study of twenty forested plots, and performed a deer exclosure experiment. We found that *I. ricinus* density was on average 71–99% lower in plots without deer than in plots with deer, depending on the life stage, but in plots that had deer, we found no correlation of *I. ricinus* density with deer availability (Fig. 2). Excluding deer for two years from small (< 1 ha) forest plots using a fence decreased nymph and adult densities of *I. ricinus* with 66% and 32%, respectively (Fig. 3), hence fencing reduced *I. ricinus* density even at small scales. We therefore conclude that deer are essential hosts for *I. ricinus* in this system, but that *I. ricinus* density is not driven solely by the availability of deer.

Our finding that the number of questing ticks of all three life stages was lower in sites where deer were absent (Fig. 2) is in agreement with two earlier studies on North American tick species of the *I. ricinus* complex, which each found a dramatic decrease in tick densities after elimination of deer populations [11, 29]. Especially larvae were much less abundant at the two plots where deer were naturally absent. This was despite the fact that alternative hosts for adult ticks were available in the form of European hare (*Lepus europaeus*), European hedgehog (*Erinaceus europaeus*), European pine marten (*Martes martes*), or red fox (*Vulpes vulpes*), as indicated by camera-trap data. Although all of these host species can support feeding adult *I. ricinus* [8], their presence appeared insufficient for sustaining a large *I. ricinus* population. Therefore, our data do not suggest that these species are important hosts for adult *I. ricinus*. The difference in density was smaller, but still substantial, for nymphs and adults. This can be explained by immigration of larvae and nymphs into a site while feeding on a host [30]. Our results imply that only complete elimination of deer substantially reduces densities of questing *I. ricinus*, even in the presence of alternative hosts for the adult stage.

We found that exclusion of deer from small forest plots (< 1 ha) caused a substantial reduction of *I. ricinus* in these plots, as much as 66% for nymphs. This finding agrees with Gilbert et al. [9], who found a strong decrease in the density of *I. ricinus* nymphs in both large and small (< 1 ha) exclosures. However, our findings are not in line with a meta-analysis by Perkins et al. [13] who found that exclosures > 2.5 ha were needed to reduce densities of *I. scapularis* and *Amblyomma americanum*. Perkins et al. [13] also studied the number of *I. ricinus* parasitizing on small rodents in two small (< 1 ha) forest plots in Italy, and found no difference in larval burden, and an increase in nymphal burden on small rodents in exclosures compared to control plots [13], suggesting that small exclosures did not effectively reduce *I. ricinus* densities in that system. The discrepancy may be explained by the fact that Perkins et al. [13] included studies in which effects of exclosures on tick densities were measured one year after placement. Failure of adult ticks to find hosts should cause the number of larvae to collapse within one year, but should cause the number of nymphs to collapse only after two to three years [31]. It is even possible that the number of questing ticks increases initially after exclosure placement due to the lower likelihood of finding a host, as we observed in 2013 for adult *I. ricinus* (Fig. 3). Therefore, we conclude that small exclosures of approximately 1 ha can already reduce densities of questing *I. ricinus*.

Van Buskirk & Ostfeld [16] predicted that the relationship between deer density and questing nymphal *Ixodes* density should follow a threshold relationship when immature stages mainly feed on other host species. Our results fully support this prediction, as *I. ricinus* density increased strongly with deer presence but not with deer abundance. Only the plot with the lowest availability of deer (Herperduin) had tick densities similar to those in plots without deer (Fig. 2), suggesting that the threshold for deer passage rate (our measure of deer abundance) lay somewhere between 0.012 and 0.018 m^-1^·d^-1^. These results agree with a study on *I. scapularis* in which large variation in deer density did not result in changes in questing nymphal density, while there was a weak correlation with larval density [32]. Such a threshold relationship, which was already suggested in 1988 by Wilson et al. [29], can explain equivocal effects of deer culling on densities of ticks in the *I. ricinus* complex [12].

Our findings are in contrast to two studies in continental Europe that found a linear relationship [17] and a parabolic relationship [33] between deer density and *I. ricinus* density at larger spatial scales. This difference could be due to the use of census estimates of deer abundance across larger regions by both Sprong et al. [17] and Tagliapietra et al. [33]. As deer do not use the landscape homogenously (e.g. [34]), census estimates of larger regions could mask a threshold relationship at smaller spatial scales due to presence or absence of deer at sites where ticks were dragged, resulting in a linear relationship due to averaging at the larger spatial scale.

Our results are also in disagreement with four studies in Scotland [9, 35–37] that found a linear correlation between *I. ricinus* density and estimates of deer density at a small spatial scale. First, this could be because these studies used dung counts to determine the local availability of deer to ticks. Unlike camera trapping, which only records deer while moving, dung counts account for deer bedding in an area while ruminating, which represents additional time during which ticks may drop off. However, dung counts have their own limitations. It is often difficult to identify species based on faeces [38]. Furthermore, defecation rates and decay rates of dung might differ between areas, resulting in biased estimates of abundance. Finally, camera trapping also captures hosts that cannot be surveyed with dung counts. We therefore think that camera trapping provides a better estimate of host availability to ticks than do dung counts.

Secondly, the discrepancy between the Scottish studies and our result could be due to the fact that deer seem to be more important as hosts for the sub-adult stages of *I. ricinus* in Scotland [39], compared to the Netherlands [8]. When deer are the most important host species for all stages of *I. ricinus*, a linear relationship might be expected between deer density and tick density. However, when other hosts, such as small mammals are available, deer might not play an important role as hosts for the immature stages of *I. ricinus* [8], resulting in a threshold relationship [16]. This shows that even studies on the same tick species cannot be directly translated from one study to the next, and that the availability of different host species for the immature life stages of *I. ricinus* should be taken into account when estimating the impact of deer management on tick densities.

## Conclusions

Our findings have major implications for the possibility of deer management for the control of *Ixodes ricinus* densities in multiple-host systems. We argue that reduction of deer densities in these systems may not reduce *I. ricinus* density, unless deer are completely eliminated. Moreover, locally low deer densities may unintentionally enhance dispersal of deer from other areas, which may quickly increase tick densities again [15]. Thus, deer culling does not seem an effective strategy to reduce *I. ricinus* density, except in situations in which deer are the single most important hosts for immature as well as adult stages, as e.g. in Scotland [9]. In contrast, excluding deer by fencing the area can be used to successfully decrease densities of *I. ricinus*, even at small spatial scales. Fencing could for example be used to reduce the risk of acquiring a tick bite in areas with high recreational pressure, such as campsites and playgrounds.

## Additional files

Additional file 1: Table S1. Characteristics and sampling effort (camera days) of the research sites of the cross-sectional study. (DOCX 18 kb)

Additional file 2: Table S2. All data used for the analysis of the cross-sectional study. **Table S3.** All data used for the analysis of the experiment. (XLSX 14 kb)

## Abbreviation

GLMM: Generalized Linear Mixed Model
