# Supplementary material for: Deer presence rather than abundance determines the population density of the sheep tick, Ixodes ricinus, in Dutch forests
Source: Parasit Vectors. 2017 Sep 19;10:433. doi: 10.1186/s13071-017-2370-7 (PMC5606071; doi:10.1186/s13071-017-2370-7)
Supplement: Supplementary file 1 — Characteristics and sampling effort (camera days) of the research sites of the cross-sectional study. (DOCX 18 kb) [file 13071_2017_2370_MOESM1_ESM.docx]

**Additional file 1: Table S1.** Characteristics and sampling effort (camera days) of the research sites of the cross-sectional study.

| Site | Habitat^a^ | Shrub layer^b^ | Undergrowth vegetation^c^ | Year | Coordinates^d^ of the plot | No. camera trapping days |
| --- | --- | --- | --- | --- | --- | --- |
| Amsterdamse Waterleiding Duinen | Mixed forest | *Crataegus monogyna* | *Calamagrostis epigejos* | 2014 | 52°20’36’’N 4°33’58’’E | 492 |
| Bergherbos | Mixed forest | *Rhamnus frangula* | *Deschampsia flexuosa* | 2013 | 51°55’14’’N 6°14’30’’E | 504 |
| Buunderkamp | Scots pine forest | *Rhamnus frangula* | *Vaccinium myrtillus* | 2013 | 52°00’56’’N 5°44’50’’E | 504 |
| Duin en Kruidberg | Mixed forest | *Crataegus monogyna* | *Calamagrostis epigejos* | 2013 | 52°26’16’’N 4°36’18’’E | 504 |
| Deelerwoud | Scots pine forest | *Betula pendula* | *Vaccinium myrtillus* | 2014 | 52°05’51’’N 5°56’42’’E | 504 |
| Enkhout | Scots pine forest | *Betula pendula* | *Vaccinium myrtillus* | 2013 | 52°16’25’’N 5°54’49’’E | 495 / 504^e^ |
| Herperduin | Mixed forest | *Rhamnus frangula* | *Molinia caerulea* | 2014 | 51°45’33’’N 5°36’53’’E | 504 |
| Halfmijl | Mixed forest | *Rhamnus frangula* | *Molinia caerulea* | 2013 | 51°25’23’’N 5°19’09’’E | 504 |
| Kremboong | Pedunculate oak forest | *Rhamnus frangula* | *Dryopteris dilatata* | 2013 | 52°45’13’’N 6°31’16’’E | 504 |
| Maashorst | Mixed forest | *Rhamnus frangula* | *Deschampsia flexuosa* | 2014 | 51°42’44’’N 5°35’24’’E | 504 |
| Pettemerduin | Pedunculate oak forest | *Prunus serotina* | *Polypodium vulgare* | 2014 | 52°46’33’’N 4°40’19’’E | 499 |
| Planken Wambuis | Scots pine forest | *Pinus sylvestris* | *Vaccinium myrtillus* | 2013 | 52°01’54’’N 5°48’36’’E | 441 |
| Rheebruggen | Pedunculate oak forest | *Rhamnus frangula* | *Dryopteris dilatata* | 2014 | 52°46’60’’N 6°17’44’’E | 504 |
| Schoorlse Duinen | Mixed forest | *Quercus robur* | *Molinia caerulea* | 2013 | 52°41’47’’N 4°40’01’’E | 504 |
| Stameren | Mixed forest | *Prunus serotina* | *Deschampsia flexuosa* | 2014 | 52°03’38’’N 5°21’01’’E | 486 |
| Valenberg | Scots pine forest | *Pinus sylvestris* | *Vaccinium myrtillus* | 2014 | 52°15’33’’N 5°48’47’’E | 391 |
| Vijverhof | Mixed forest | *Sorbus aucuparia* | *Deschampsia flexuosa* | 2013 | 52°09’43’’N 5°13’43’’E | 507 |
| Vledderhof | Pedunculate oak forest | *Rhamnus frangula* | *Dryopteris dilatata* | 2014 | 52°52’46’’N 6°14’25’’E | 504 |
| Zwanemeerbos | Pedunculate oak forest | *Sorbus aucuparia* | *Pteridium aquilinum* | 2013 | 53°00’46’’N 6°45’19’’E | 504 |

^a^ Forest plots were dominated by Pedunculate oak (*Quercus robur*), Scots pine (*Pinus sylvestris*), or a combination of both (mixed forest).

^b^ The given plant species was the most dominant species in the shrub layer.

^c^ The given plant species was the most dominant species in the herbaceous layer.

^d^ Coordinates as measured with a handheld GPS (Garmin eTrex 20) in the middle of the plot.

^e^ The number of camera trapping days outside of the exclosure (left) and inside the exclosure (right).
